# Biview Learning for Human Posture Segmentation from 3D Points Cloud

**DOI:** 10.1371/journal.pone.0085811

**Published:** 2014-01-20

**Authors:** Maoying Qiao, Jun Cheng, Wei Bian, Dacheng Tao

**Affiliations:** 1 Centre for Quantum Computation and Intelligent Systems, Faculty of Engineering and Information Technology, University of Technology, Sydney, New South Wales, Australia; 2 Guangdong Provincial Key Laboratory of Robotics and Intelligent System, Shenzhen Institutes of Advanced Technology, Chinese Academy of Sciences, Shenzhen, Guangdong Province, China; 3 The Chinese University of Hong Kong, Hong Kong, China; University of Ulm, Germany

## Abstract

Posture segmentation plays an essential role in human motion analysis. The state-of-the-art method extracts sufficiently high-dimensional features from 3D depth images for each 3D point and learns an efficient body part classifier. However, high-dimensional features are memory-consuming and difficult to handle on large-scale training dataset. In this paper, we propose an efficient two-stage dimension reduction scheme, termed biview learning, to encode two independent views which are depth-difference features (DDF) and relative position features (RPF). Biview learning explores the complementary property of DDF and RPF, and uses two stages to learn a compact yet comprehensive low-dimensional feature space for posture segmentation. In the first stage, discriminative locality alignment (DLA) is applied to the high-dimensional DDF to learn a discriminative low-dimensional representation. In the second stage, canonical correlation analysis (CCA) is used to explore the complementary property of RPF and the dimensionality reduced DDF. Finally, we train a support vector machine (SVM) over the output of CCA. We carefully validate the effectiveness of DLA and CCA utilized in the two-stage scheme on our 3D human points cloud dataset. Experimental results show that the proposed biview learning scheme significantly outperforms the state-of-the-art method for human posture segmentation.

## Introduction

Posture segmentation, i.e. partitioning a human body into semantic parts (such as, torso and limbs), is an indispensable step in human motion analysis [1], [2], among various practical applications, from security surveillance (abnormal detection, human activities analysis), interfaces to games (seen in EyeToy [3]), virtual reality and/or human-computer interfaces, and to video annotation. However, inferring the pose of a highly articulated object is considerably challenging due to its inherent complexity caused by the changing of body pose and the diversity of shape and appearance of individuals. Posture segmentation has been a highly active research area for decades.

Early studies on human posture segmentation were mainly based on conventional intensity images. There are several hurdles to overcome in this direction of study, including (1) complex environment situation, such as varied textures, lighting conditions, scales, (2) ambiguity caused by missing depth information, such as self-occluding problems, and (3) highly computational cost. Many works run up against one or more of these difficulties. G. Mori *et al.* [4] match up the test image with the stored exemplars using the shape context descriptor. It falls into an embarrassment that more exemplars containing complete appearance are required to get a high accuracy while less exemplars are desired to achieve an efficient matching. L. Pishchulin *et al.* [5] develop a complete and controlled database to manage the appearance, shape and pose variations. P. Felzenszwalb *et al.* [6] utilize the pictorial structure models, which separately represent appearance of each part, to reduce the large variation in shape and photometric information in each object class. Z. Tu [7] proposes to learn the context information by a discriminative [8] probability maps on local image patches[9]. Combining the learned context information with the original image patches, it trains an integrated low-level context model to get the human body configuration. In the test stage, it typically takes about 30∼70 seconds per image of size around 300×200, which is far from the requirement in real-time applications. In [10], C. Bregler and J. Malik take the problem of tracking humans as a differential motion estimation using the product of exponential maps and twist motions. Given a close initial pose, the algorithm would converge correctly and quickly. However, the performance of the algorithm depends heavily on the initialization.

The emerging of depth cameras stimulates new methodologies for human posture segmentation, which overcomes the abovementioned first two weaknesses of intensity image based methods. D. Simon *et al.* [11] utilize both conventional range sensors and CMU high speed VLSI range sensor to capture model and real-time range data of the rigid object respectively. Then, iterative closest point (ICP) algorithm, which tries to rigidly transform one points cloud to another by minimizing corresponding points' distances, is performed for real-time pose tracking and estimation. The works [12], [13] also apply ICP algorithm to depth data to track an initialized skeleton. Besides, the point cloud library (PCL) [14] provides open source implementation of ICP algorithm. Although those pose tracking and estimation methods accomplished with ICP can satisfy the real-time requirement, they need to be re-initialized quickly because the tracking is not robust due to fast human motion and accumulated errors.

Along the launch of Kinect [15], 3D points cloud can be processed at consuming level [16]. J. Shotton *et al.* [17] introduce the core of points cloud handling component of Kinect gaming platform. They obtain the 3D locations of skeleton joints from human point clouds through three steps. First, high-dimensional features based on depth information for each pixel are extracted from the depth images. Second, randomized decision forests are trained to label each pixel which body part it belongs to. Finally, joint positions are proposed from the body part recognition result by local model-finding technique based on mean shift [18]. However, high dimensionality (2000-dimension features in experimental setting of [17]) is a severe deficiency. To handle this disadvantage, [17] proposes to use randomized decision forests to select effective dimensions preserving most useful group information. Even though the assumption that body joint locations are independent from each other which is only approximately true in practice [19], the algorithm achieves encouraging accuracy. Furthermore, M. Sun *et al.* [19] try to exploit the dependency relationships among body parts through global prior knowledge, i.e. torso orientation and/or person height, based on the work of regression forests [20]. More techniques to deal with points cloud are listed in PCL [14], such as min-cut based segmentation which makes a binary segmentation of the points cloud, as well as several features extracted from points cloud: Fast Point Feature Histograms (FPFH), normals based segmentation, surface normals estimation in points cloud. Our previous work [21], which is based on surface normals, attempts to solve posture segmentation from a different aspect. It constructs human body manifold space from 3D position features. In addition, it integrates surface normal features as constraints into the final spectral space to get more meaningful segmentation results. However, two eigen-decomposition operations on large matrix prevent the algorithm from real-time applications. All of these state-of-art features are less popular than the feature proposed in [17] in terms of highly computational efficiency as well as sufficient information for categorizing pixels into different body parts. However, high-dimensional features are not preferred [22] for most posture segmentation techniques. In this paper, we propose a novel biview learning algorithm for human posture segmentation from 3D points cloud provided by Kinect. Dimensionality reduction is a crucial way to deal with the “curse of dimensionality” [23]. Here, we apply the recently proposed discriminative locality alignment (DLA) algorithm [23]–[25] to transform the high-dimensional depth different features (DDF) to a low-dimensional representation which reveals the manifold distribution of depth pixels and owns more discriminative ability. To generalize the learned feature space from training set, we introduce unsupervised 3D relative position feature (RPF) for each depth pixel, which is another view independent of DDF, and employ biview canonical correlation analysis (CCA) [26]–[28] to unify those two views. Therefore, we can further reduce the dimensionality of the dimension reduced DDF by maintaining only the strongly correlated directions between the two views. Finally, we train a multi-class SVM [29]–[32] to accomplish the task of posture segmentation.

We specifically represent our proposed framework step by step in Section 2. In Section 3, first, we verify the performance of the DLA with our dataset, in terms of effectiveness of both recognition rate and dimensionality reduction, in comparison with other popular dimension reduction algorithms, such as PCA, LDA, etc. Then, we validate the effectiveness of our two-stage dimension reduction scheme for posture segmentation. Conclusions and discussions are given in Section 4.

## Method Overview

(We received the formal written waiver for the ethic issues of the collected data. The ethics committees of Shenzhen Institutes of Advanced Technology approve this consent procedure. There is no problem to make the data used in the paper publicly available. We didn't conduct research outside of our country of residence. All participants provide their written informed consent to participate in this study.)

**Figure 1:**
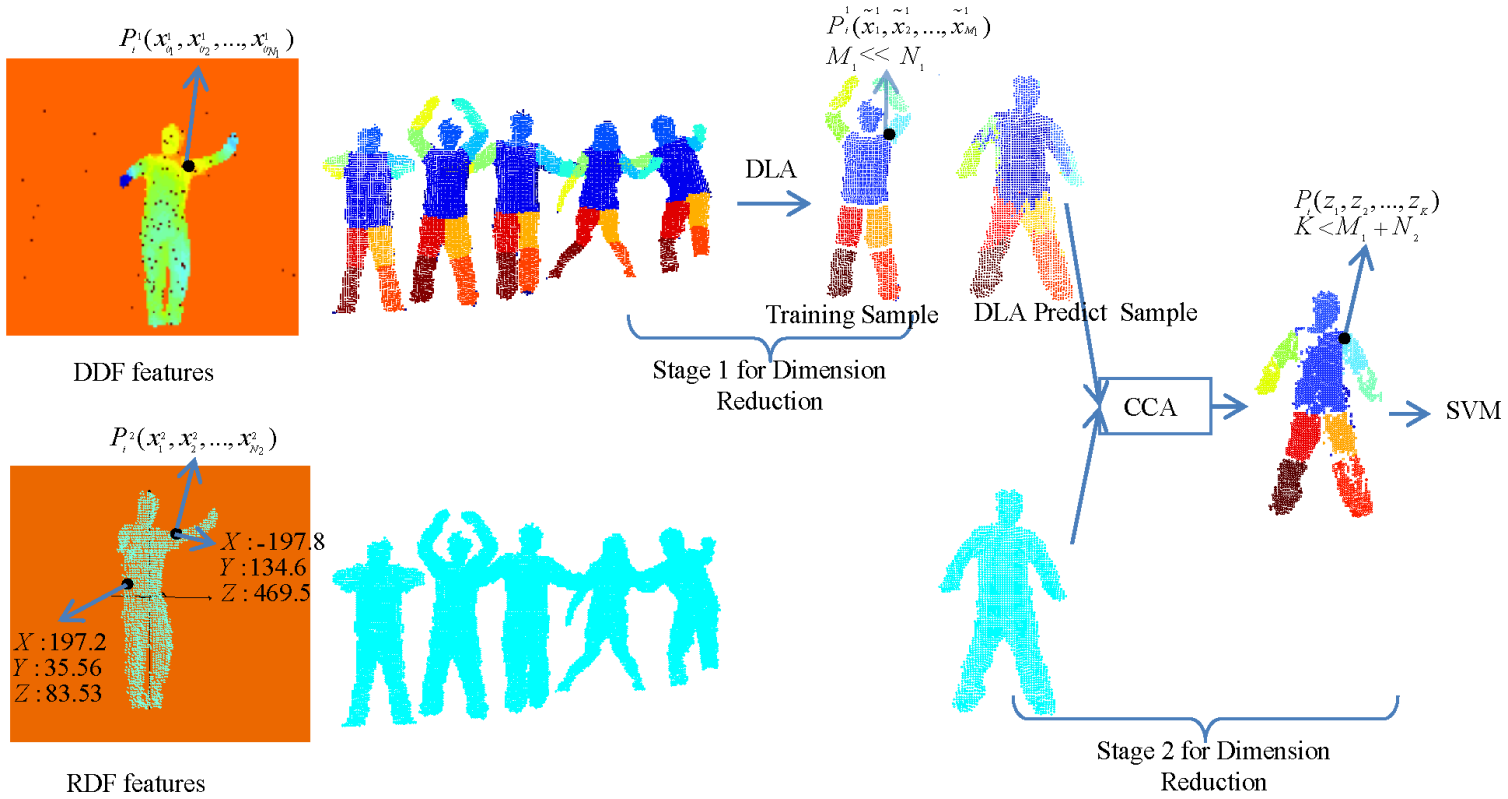
Biview learning framework for human posture segmentation. The first row demonstrates extracted original high-dimensional depth features (DDF), and then using the training data, we apply DLA as our first stage for dimension reduction to obtain more discriminative features. From the training sample, apparently, the points on different body parts are separated with high margins. The second row demonstrates the extracted unsupervised relative position features. By CCA, it tries to explore complement information, namely, using the unsupervised RPF adjusts the overfitting of the learned features while using learned DDF features to introduce more discriminative ability. Finally, the k-d features (k is much less than the dimensions of DDF) are inputted to train a traditional SVM classifier.

Given *N* 3D human points 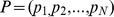 appearing both in the 2D depth images 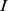 and 3D points cloud, and their corresponding labels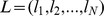, where 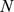
is the total number of human points and each label 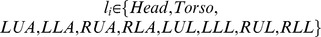. (LUA stands for Left Upper Arm, LLA for Left Lower Arm, RUA for Right Upper Arm, RLA for Right Lower Arm, LUL for Left Upper Leg, LLL for Left Lower Leg, RUL for Right Upper Leg, and RLL for Right Lower Leg.) In this paper, through biview learning, we aim to find a low-dimensional representation 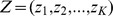 from two different views, i.e., globally discriminative structure of point expressed as high-dimensional depth difference features (DDF) 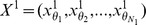 and local 3D geometric manifold coordinates of point represented by the relative position features (RPF) 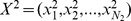, for posture segmentation, where 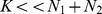. The dimensions of DDF and RPF are 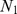 and 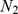 respectively, and here 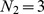 . Fig. 1 illustrates the proposed biview learning framework of the two-stage dimension reduction scheme for posture segmentation. First, we extract DDF and RPF from depth images. Second, DLA is applied in Stage 1 for dimension reduction. Then, the learned low-dimensional DDF feature space is regularized by unsupervised 3D RPF via CCA, which is considered as Stage 2 for dimension reduction. Finally, SVM is trained to complete the task of human posture segmentation. Before we explain each step in detail in the following subsections, we list all of notions throughout the paper in Table 1.

**Table 1:** Important notations used in the paper.

Notation	Description
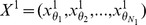	high-dimensional DDF
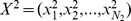	relative position features (RPF)
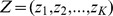	final low-dimensional representation
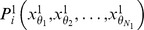	DDF features for point 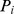
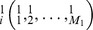	low-dimensional space for 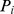
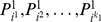	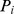 's 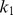 nearest neighbors with the same class label
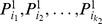	Point 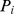 's 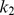 nearest neighbors with the same class label
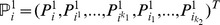	local patch of 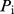 in the original high-dimensional space
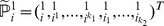	local patch of 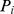 in the low-dimensional space

### Stage 1 for dimension reduction using DLA

#### Depth difference features (DDF)

We adapt the depth difference feature (DDF) [18] for each human point, which is defined as below
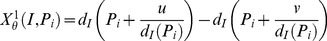
(1)where 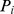 is one point in depth image 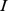 , 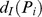
describes the depth of point 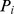 , and parameter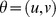 containing offsets 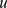 and 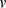 demonstrates two point 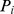-centered locations in the depth image. The normalization of the offsets by 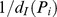 ensures that the features are 3D translation invariant, which overcomes the scale-variant problem in the traditional images. As defined, the DDF for each point can be computed by five simple operations (two divisions, two additions, one subtraction), which is computationally efficient.

**Figure 2:**
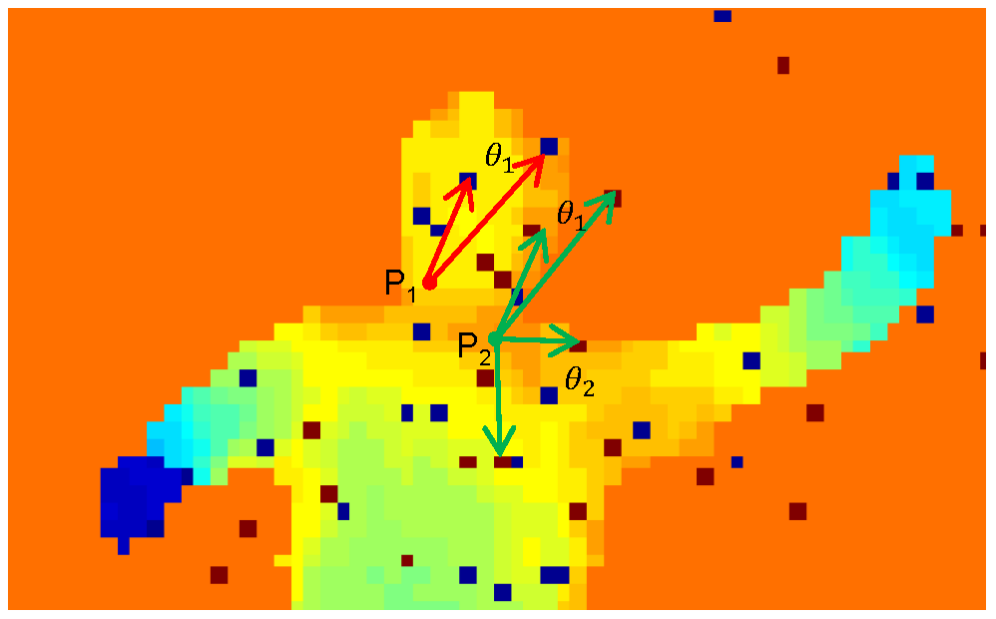
Denoting of depth different features (DDF). Parameter 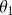 for point 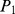 is geometrically defined by two red arrows, 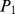-centric, corresponding to pairwise offsets u and v. The depth difference for (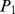,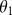) is the absolute value of depth difference between two points located at the arrowheads. Apparently, the absolute values of depth differences for (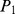,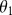), (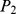, 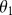), (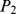,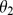) are unequal.

We illustrate parameters
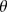
in Fig. 2 to get more straightforward sense. As shown in Fig. 2, parameter 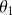 for point 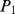
is geometrically defined by two red arrows, 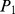-centric, corresponding to pairwise offsets 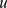 and 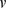. We take two different points, 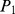 located in the head and 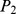 located in the torso, as an example to show DDF's effectiveness. First, both 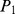 and 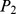 are assigned with the same parameter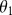, but they have different DDF responses, namely, 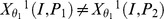. This reveals small discriminative power of DDF for posture segmentation. Second, combining another different parameter 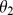 for point 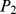 with parameter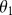, apparently, 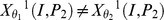 and we can get different depth distribution among neighbors of 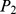. By combining more DDF responses with different offset parameters for each point into a high-dimensional DDF features, it tends to recovery global depth manifold and provide strongly discriminative signals about which body part the point belongs to. In our setting, 500 pairwise offset parameters are randomly predefined for each human body point. Demonstrated by dark red squares (dark blue squares) in Fig. 2, the high-dimensional DDF features uniquely determine the depth characteristic of 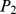
(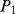) in the whole depth image, which is crucial information for labeling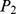.

At last, we complete our DDF introduction by explaining lower and upper limits for the depth difference. The depth difference for pairwise offsets (u, v) ranging from 0, which indicates two points locate in the same depth plane, to 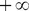, which expresses the depth difference between background points or between body points and background points. Usually, the maximum depth difference between two body points is around 1 m.

However, the high-dimensional features are hard to deal with for most algorithms. This motivates us to employ DLA, a state-of-the-art dimension reduction algorithm, to transform the DDF features to low-dimensional representations. This reveals the intrinsic structure of data distribution meanwhile preserves discriminative information.

#### Review of DLA

**Figure 3:**
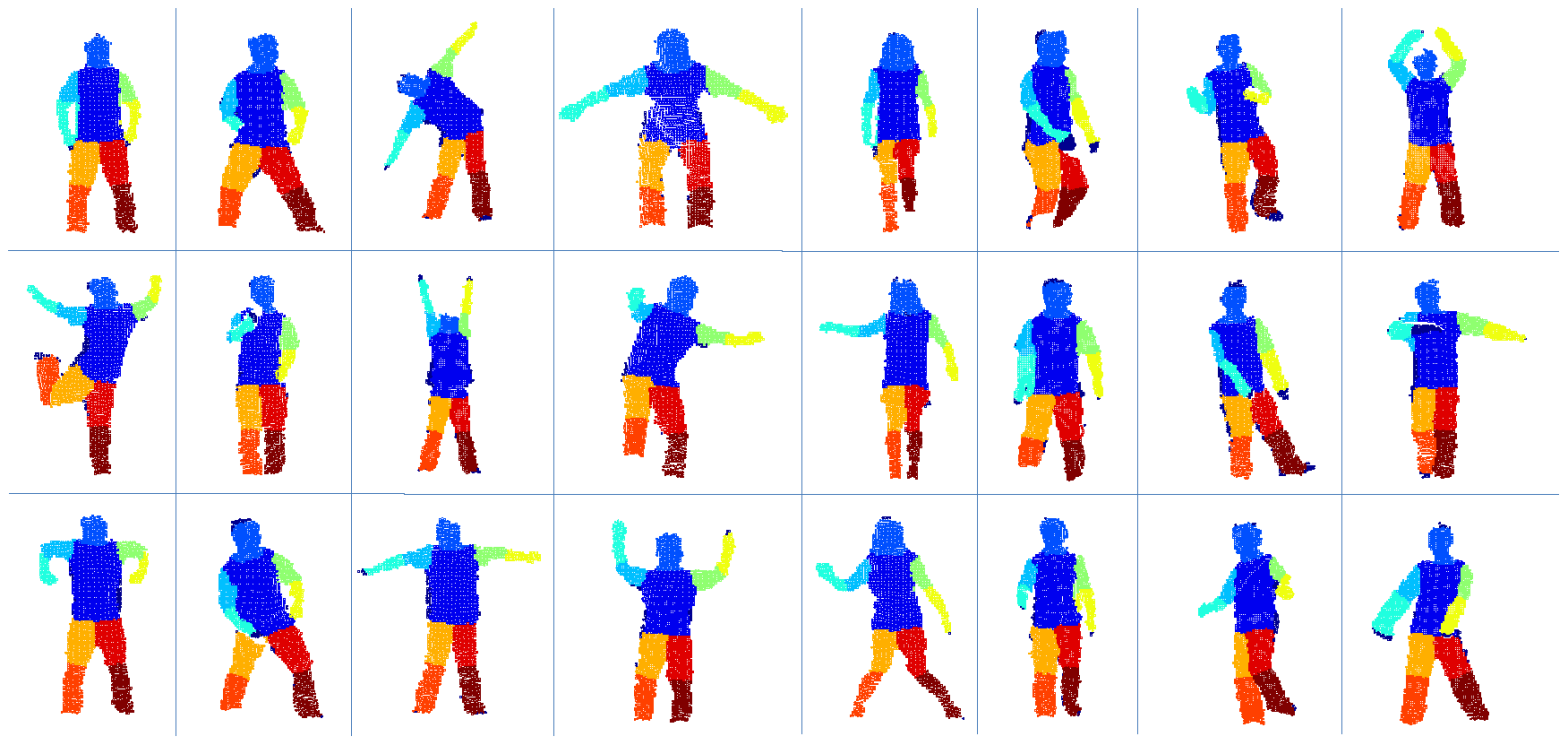
Sample frames for different persons (columns) performing different activities (rows). Eight persons with variance of height, weight, gender are selected from our training dataset (from our labeled dataset) and three frames of different activities per person are shown. From these frames, we can see that our dataset contains a variety of daily activity frames.

Discriminative Locality Alignment (DLA) is a dimension reduction technique, designed in particular to preserve the local discriminative information of data distribution. In the context of posture segmentation, suppose we have a set of labeled training data, e.g., 24 samples are shown in Fig. 3, we apply DLA to obtain a low-dimensional representation of the DDF feature. Specifically, given a point 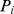 from the training set, whose DDF feature is 
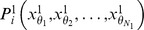
, we find its 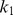 nearest neighbors from the training data points with the same class label, i.e., 
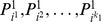
, as well as its 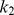 nearest neighbors with different class labels, i.e., 
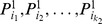
 . We use these nearest neighbors to construct a local patch for each point 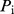, 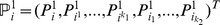 . The point 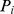 in the low-dimensional space is presented as 
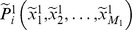
, and correspondingly, the local patch of 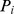 is: 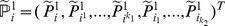. We emphasize that 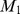 is the dimensions of the low-dimensional representation and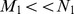.

The core idea of DLA is that it tries to find a low-dimensional representation to make the points from the same body part closer while to keep the points from different parts further [20], by exploiting both local geometry and discriminative information. DLA is modeled as the following objective functions respectively for the given point 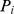


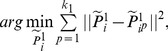
(2)




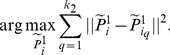
(3)


Combining within-class measures Eq.(2) with between-class measures Eq.(3) by a scaling factor 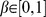, we get
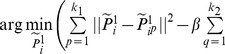
(4)


Here, 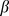 tries to keep the two measurements in balance. There are two factors that can cause the imbalance. First, the numbers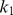 and 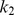, of the same-class and different-class nearest neighbors are unequal, and usually it holds 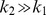 in the training set. Second, for most of the points scattered in the human body, the distance from point 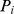 to the same-class nearest neighbors are usually much smaller than the distance to the different-class nearest neighbors. We use the scaling factor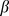, ranging in [0, 1], to adjust the tradeoff between the two measurements. For experiments, we simply set 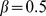. Then we select values of 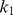 and 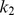 by adopting the same procedure used in [23]. 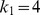 and 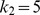 are finally settings for our experiments.

By introducing the coefficients vector 
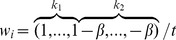
, we integrate the two parts into a uniform format
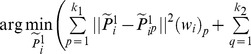
(5)


Finally, by organizing the elements of the 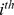 local patch into a matrix, we get the objective function
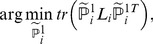
(6)where
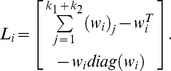
(7)


Assuming the local patch of 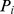 : 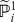 is selected from a global coordinate, i.e., 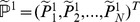, where 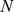
is the total number of training points, namely,
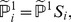
(8)where 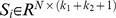 is the index matrix of 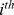 local patch. Then, the whole DLA model is given by
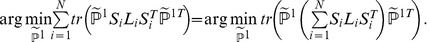
(9)


We assume that the matrix 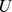 projecting the dataset from the original high-dimensional space to the low-dimensional representation is linear and orthogonal; then, the optimization problem is transformed as
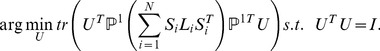
(10)where 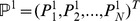 is a global coordinate of the original high-dimensional space.

The optimal solution of (10) is given by eigen-decomposition,
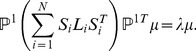
(11)


To get 
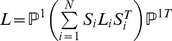
 , we can directly compute the summation 
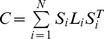
 , and then do matrix multiplication. However, it is really memory-consuming when the size of training set 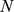 is large, as the size of matrix 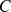 is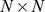. So, here, we put 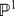 into the summation, and firstly compute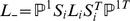, whose size is 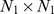
(the dimensions of DDF features) for each training point, then iteratively do the sum operator. In this manner, we just trade more training time for less memory requirement. Besides, we can also implement it in distributed computers efficiently.

After performing DLA, we learn a low-dimensional representation which preserves both discrimination information and intrinsic local geometry for the training data. However, the dimensionality reduced DDF features are learned from the training data, which are only a small fraction of the whole dataset. This makes the low-dimensional representation not well-generalized for the test data. We need to explore more generic information to regulate the learned low-dimensional representation so as to obtain better generalization ability.

### Stage 2 for dimension reduction using CCA

#### Relative position features (RPF)

In addition to the view of globally discriminative power provided by the DLA-reduced DDF, we try to hold the 2D human surface manifold embedding in the 3D real-world coordinates by simply employing the 3D coordinate values. We make use of the barycenter of human body points as the origin point of the 3D coordinates and translate human body points from real-world coordinates to the barycenter coordinate. We term the new coordinate values for each point 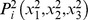 as relative position features (RPF). On the one hand, RPF is directly obtained from the original data and thus has no extra computational cost. On the other hand, RPF straightforwardly constructs the human surface manifold, an intrinsic view of human body, and thus is useful for partitioning the articulated human body parts.

We ultimately try to get a representation with both strong discriminative power and better generalization ability. In particular, the unsupervised manifold information, i.e., RPF, improves the generalization ability, meanwhile the supervised characteristic of DDF helps to extract discriminative information from RPF. Two views – the globally discriminative view provided by DLA-reduced DDF and the local manifold view with more generalization ability provided by RPF – should be combined by an effective strategy.

Both canonical correlation analysis (CCA) and partial least squares (PLS) [33] try to find the most correlated directions between two different spaces. However, PLS performs well in the situation that one feature representation is treated as regressor and the other is as response. It does not fit to our situation well. In contrast, CCA is preferable since it can retain multiple projections for each view, and then a joint feature representation can be obtained. Additionally, the first few correlated directions of CCA usually hold the majority of relevant information between the dimensionality reduced DDF and RPF, which indicates that we get an even lower-dimensional representation.

#### Review of CCA

Canonical Correlation Analysis (CCA) tries to linearly project the two different views from their individual spaces to their most correlated lower-dimensional subspace, which is a special case of popular multiview analysis [34]–[42] Let 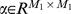 and 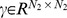 be the projection matrices for the learned DDF 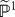 and unsupervised RPF 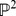 respectively, 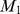 and 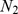 are maximum correlated dimensions. The correlation coefficient between the two projected variables is defined as:
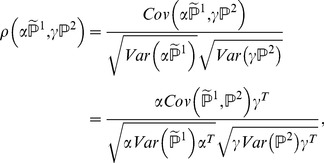
(12)where 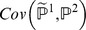 is the covariance between 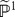 and 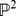, 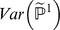 and 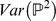 are variances of 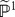 and 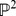 respectively. Suppose 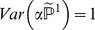 and 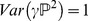 , CCA can be solved by the optimization below
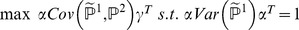
(13)


The optimal solution of (13) is given by the Singular Value Decomposition (SVD) on 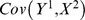 [33]

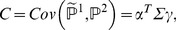
(14)where 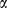 is the left singular matrix of 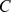, the diagonal entries of 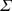 is the singular values, and 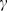 is the right singular matrix. As the left and the right singular vectors correspond to the maximum singular value project the original variables 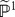 and 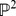 into the most correlated subspace, we concatenate first 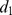 columns of 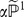 and 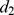 columns of 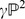 as our final low-dimensional representation 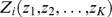, where 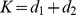.

### SVM for human posture segmentation

Based on the low-dimensional representation, we finally train a multi-class SVM classifier to partition the human body points into different semantic parts. SVM [29] is based on structural risk minimization inductive principle and tries to divide samples in separate categories by a clear margin as wide as possible in a high-dimensional space projected by a kernel function. There are two advantages for training SVM to predict the test set human points. First, SVM avoids the curse of dimensionality but keeps power of linear separability. Second, the solution based support vectors which determine the parameters of the discriminant function are sparse. We can do predictions depending only on a subset of the training data points rather than all of them. Obviously, it is helpful for real-time applications.

We apply LIBSVM [30], [31] to train our multiclass SVM by building 10 binary SVMs through the one-against-the-rest strategy. New instance is classfied as the class whose corresponding classifier outputs the highest score. The kernel function we employ here is the Gaussian redial basis function (RBF): 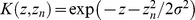, as RBF-SVM is capable of both low error rate for training set and well-generalization for testing set once given an appropriate variance 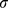. We use five-fold cross validation to select the optimal value for 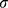.

## Experimental Results

We collect our database utilizing Kinect sensor. We assume that four persons are trying to control the human-computer interactions. Usually four different persons face the sensor, stand nearly 1.2 m away from the Kinect sensor and do random activities as they want. They can twist their torso within ±30 degrees during their activities. If more perspectives are performed, more strategies should be applied as our human body are symmetrical which is hard to be identified under our framework. Each person performs different activities, and contributes to balanced pose dataset with four 5-min videos with poses of turning around, left-lifting, squatting, arm-carrying. Finally a dataset containing around 12,000 frames is constructed. First, we remove points of background and ground floor. Then, we manually label each point with auxiliary of joints' positions outputted by Kinect. We implemented a software modular to assist in blockily labeling points with the initialized joint position. Even so, labeling the points is still a labor intensive work and each frame takes 30 seconds to be labeled on average. Besides, we allow several outliers to exist to build the sense of robustness of our algorithm. We randomly choose 70% of frames as the training set and use the remaining 30% as the test set. Samples of human activities in training set are shown in Fig. 3. We extract 500-dimensional DDF for each human point by generating 500 pairs of offset parameters.

In this section, we carefully validate that DLA is applicable to our dataset for dimension reduction in comparison with other supervised or unsupervised[43] dimension reduction algorithms, e.g. LDA, PCA along with classifiers of SVM and decision tree (DT) in terms of recognition rate. We also perform random forest (RF) algorithm in terms of recognition rate, which is the state-of-the-art algorithm for human points classification and incorporates dimensionality reduction functionality and classifier functionality together to achieve the human pose segmentation task. Then, we show that our biview learning algorithm with the two-stage dimensionality reduction scheme outperforms other natural schemes, such as direct views concatenation scheme, single view scheme, etc.

### DLA results

**Figure 4:**
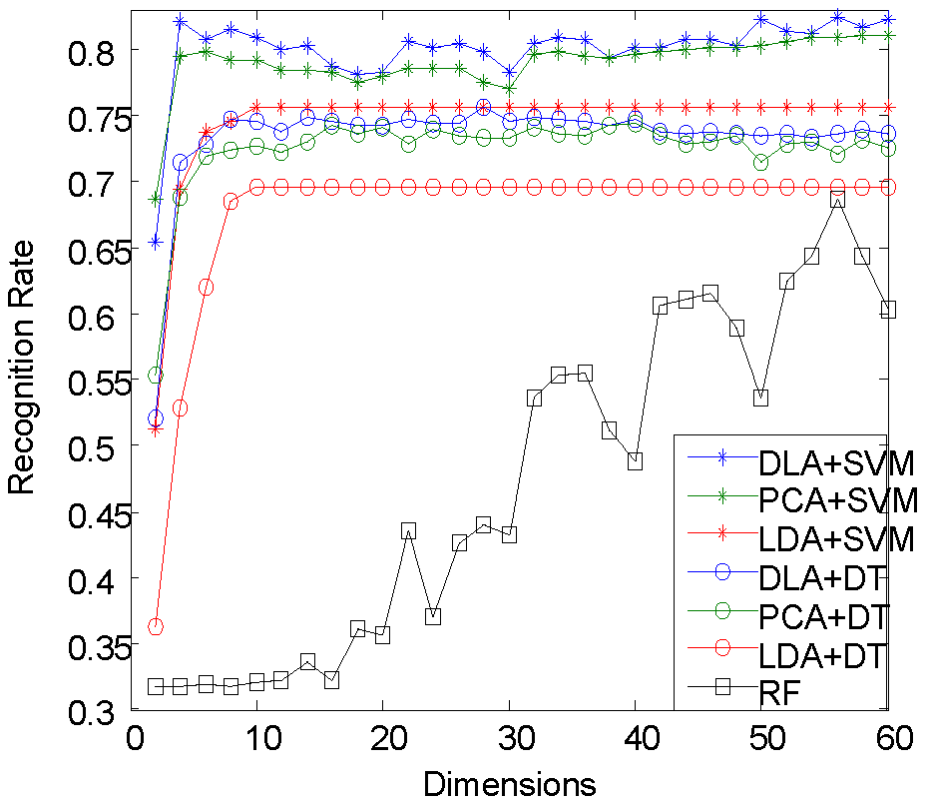
Performance for different dimension reduction algorithms and different classifiers. Seven different combinations of dimension reduction algorithms and classifiers perform differently and verify the effectiveness of DLA in our application. The overall trends of DT and SVM are similar when using the same dimension reduction algorithms. However, SVM generally outperforms DT in the low-dimensional case at nearly 6% improvement in terms of recognition rate. Concerning the dimension reduction algorithms, DLA performs better than PCA and LDA. DLA gets a higher recognition rate than unsupervised PCA regardless with the classifiers while the learned LDA performs worst as LDA tries to construct the whole data distribution by considering the within class variance and the between classes mean and thus ignores the local discriminative information which is emphasized by DLA. In our application, as the dataset of human points is really large and always varies greatly, the rough whole distribution is incapable of capturing enough discriminative information. This is verified by the result that LDA even performs badly than the unsupervised PCA. The smooth plateau part of LDA curve is caused by that the most reduced dimensions of LDA is C-1, where C is the number of classes and is 9 in our application. Comparing with Random forest (RF), we try to show the ability of our proposed schema in terms of selecting discriminative features. The result shows that the recognition rate of RF is even lower than DT with the low-dimensional features, e.g., supervised LDA features, DLA features and unsupervised PCA features.

To validate the effectiveness of DLA for our application, we conduct experiments of comparing DLA with other two typical dimension reduction algorithms on DDF in terms of the recognition rates, i.e., PCA [44] for unsupervised dimension reduction and LDA [45] for supervised dimension reduction. We train two classifiers based on SVM and decision tree (DT) [46] for classification. In the experiment, each test frame contains around 2000 body points, and we take the average recognition rate over all test frames as final performance measurement. We select 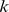 dimensions (the number of the reduced dimensions) from the low-dimensional feature space randomly, and measure all of them in each splitting node in DT to make the comparison with SVM more reasonable. The result is shown in Fig. 4.

As shown by Fig. 4, the overall trends of DT and SVM are similar when using the same dimension reduction algorithms. However, SVM generally outperforms DT in the low-dimensional case at nearly 6% improvement in terms of recognition rate.

Concerning the dimension reduction algorithms, DLA performs better than PCA and LDA. In general, features learned by supervised information own more discriminative power than the unsupervised ones. This explains why DLA gets a higher recognition rate than unsupervised PCA. Further, both as supervised methods, DLA outperforms LDA. This is because LDA tries to construct the whole data distribution by considering the within class variance and the between classes mean and thus ignores the local discriminative information but emphasized by DLA, which is especially essential for constructing the boundary between different categories. In our application, as the dataset of human points is really large and always varies greatly, the rough whole distribution is incapable of capturing enough discriminative information. This is verified by the result that LDA even performs badly than the unsupervised PCA.

We further compare our method with Random forest (RF) [47], randomly selects discriminative dimensions from the high-dimensional DDF. RF utilizes entropy information to train several decision trees (DTs) and finally obtain a decision forest. RF is employed by [17] and achieved state-of-the-art performance in human pose estimation. We train a RF with 10 DTs. Unlike the above DT, we randomly select 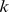 dimensions from the original DDF for each splitting node to train the RF, where 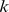 is the current reduced dimension. As shown in Fig. 4, the recognition rate of RF is even lower than DT with the low-dimensional features, e.g., supervised LDA features, DLA features and unsupervised PCA features. And DLA performs better than RF in selecting discriminative features.

### Biview learning results

**Figure 5:**
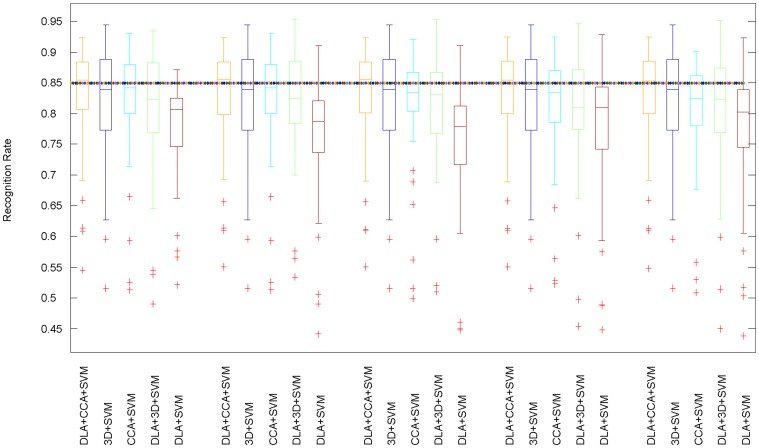
Boxplot for recognition rate vs. reduced dimensions k with different feature-integrating schemes. Other schemas (3D+SVM, DLA+SVM, CCA+SVM, DLA+3D+SVM) are compared to validate the effectiveness of our schema (DLA+CCA+SVM). In our method, we first transform the 500-d DDF into k-d low-dimensional representation; k is designed as 5, 10, 15, 20, 25. Then, by CCA, we project the k-d DDF and 3D RPF into 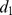
and 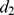 lower-dimensional representation respectively. Our proposed biview feature learning scheme achieves the best recognition rate nearly 85%. It also can be concluded that our proposed scheme is robust with respect to the reduced dimensions k. 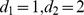 is the best setting for the highest recognition rate. Clearly, that only 3-d representation achieves highest accuracy proves the effectiveness of our scheme. Comparing with DLA+SVM and 3D+RPF, our method raises the recognition rate by 5% and 3% respectively. We can conclude that regularization established by CCA between the supervised low-dimensional DDF and unsupervised RPF takes effect for improving recognition rate. Concerning CCA+SVM, we directly try to learn correlation relationship between high-dimensional DDF and RPF. And the best setting for the highest recognition rate is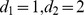. On one hand, most of the originally high-dimensional DDF have no discriminative information for labeling each human point and may introduce unexpected noise. On the other hand, CCA actually is an unsupervised method and it can also bring down the recognition rate in comparison with our schema. Finally, we analysis (DLA+3D+SVM): directly concatenating the dimensionality reduced DDF and the 3D RPF, simply joints the unsupervised and supervised information together. The manifold information embraced by RPF is complementary to the discriminative learned DDF and the recognition rate is higher than the only dimensionality reduced DDF representation. However, the representation of DLA+3D is redundant as the uncorrelated dimensions are not removed, which leads to that the accuracy is lower than our proposed one's.

To validate the effectiveness of our two-stage dimension reduction scheme (DLA+CCA+SVM), we compare it with other four feature-integrating schemes for training the SVM in terms of recognition rate:1) only one view with 3D unsupervised RPF (3D+SVM), 2) only one view with the dimensionality reduced DDF by DLA (DLA+SVM), 3) biview representation learned by CCA from high-dimensional DDF and RPF (CCA+SVM), and 4) direct concatenation of the two views of dimensionality reduced DDF and RPF (DLA+3D+SVM). The statistical performances of all these schemes are shown in the boxplot Fig. 5.The median and variability are computed from all of the test frames.

In our method (DLA+CCA+SVM), we first transform the 500-d DDF into 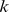-d low-dimensional representation, 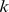 is designed as 5, 10, 15, 20, 25. Then, by CCA, we project the k-d DDF and 3D RPF into 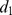 and 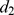 lower-dimensional representation respectively. As shown in the boxplot, our proposed biview feature learning scheme achieves the best recognition rate nearly 85%. It is also can be concluded that our proposed scheme is robust with respect to the reduced dimensions 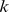. 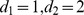 is the best setting for the highest recognition rate. Clearly, that only 3-d representation achieves highest accuracy proves the effectiveness of our dimension reduction scheme.

Comparing with the representation learned by DLA (DLA+SVM), our method raises the recognition rate by 5%. While comparing with RPF (3D+SVM), the recognition rate achieved by our method is nearly 3% higher. We conclude that the regularization of supervised low-dimensional DDF established from unsupervised 3D RPF via CCA improves the generalization and recognition rate accordingly.

Concerning the scheme of CCA+SVM, we directly try to learn correlation relationship between high-dimensional DDF and RPF. And the best setting for the highest recognition rate is 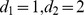. On one hand, most of the originally high-dimensional DDF have no discriminative information for labeling each human point and may introduce unexpected noise. On the other hand, CCA actually is an unsupervised method and it can also bring down the recognition rate in comparison with our proposed biview learning method.

Finally, we analysis the scheme of (DLA+3D+SVM): directly concatenating the dimensionality reduced DDF and the 3D RPF. Concatenating simply joints the unsupervised and supervised information together. On one hand, the manifold information embraced by RPF is complementary to the discriminative learned DDF and the recognition rate is higher than the only dimensionality reduced DDF representation. On the other hand, the representation of DLA+3D is redundant as the uncorrelated dimensions are not removed, which leads to that the accuracy of this scheme is lower than our proposed one's.

To Sum up, our proposed two-stage biview learning scheme achieves robustly highest recognition rate no matter how many dimensions are left comparing with other schemes. Besides, the final 3-d representation achieves as high mean value of recognition rate as other higher dimensions. This verifies the effectiveness of our proposed scheme for dimension reduction.

## Conclusion

In this paper, we have proposed a two-stage biview-learning dimension reduction scheme for human posture segmentation. First, we extract DDF and RPF from two independent views. Then, we apply DLA to learning a discriminative and low-dimensional representation from the high-dimensional DDF and take this procedure as our stage 1 for dimension reduction. Thirdly, we employ CCA to combine the two views to generalize the learned low-dimensional DDF by unsupervised RPF as well as to shape boundary of human manifold by the supervised low-dimensional DDF features. Experimental result validates the effectiveness of our proposed dimension reduction scheme. Not only our scheme achieves the highest recognition rate, but also our dimensionality reduction scheme gets an inspiring low-dimensional representation. In the future, we will capture more human activities with more persons to enlarge our dataset, on which we will measure the performance of our method to prepare it for human activity analysis applications.
